# Penile cancers without the AIDS epidemic in Cameroon

**DOI:** 10.1186/1750-9378-7-S1-P19

**Published:** 2012-04-19

**Authors:** Blaise Nkegoum

**Affiliations:** 1 University Hospital Center, Yaounde, Cameroon

## Background

Cancer of the penis is an uncommon malignancy in developed countries, But the incidence is as high as 17% of all male cancers in some undeveloped countries. The most important aetiologic factor is the presence of an intact foreskin but this is still unknown.

Cameroon is a blank area on the world cancer map because medical facilities necessary for recording cancer cases and the population data necessary for the calculation of rate are scarce or inexistent. Only 10% of malignant neoplasms are confirmed by histology.

## Methods

We described the pathological aspects of 10 cases of penile cancers observed in Cameroon, a developing country of 20.000.000 inhabitants, within a period of twenty seven years (1984-2011). Human Immunodeficient Virus (HIV) serology test was done for nine patients of this series. Human Papilloma Virus (HPV) DNA detection and typing were carried out on paraffin-embedded specimens of our cases by Polymerase Chain Reaction.

## Results

The patients aged 43 to 75 years and were circumcised. Four of the ten cases were diagnosed in 2004. HIV serology test done on 3 cases before 2004 were negative. After 2004, six patients were registered and out of these six, three came down with HIV-AIDS.

One patient has type II diabetes mellitus. All patients consulted late with metastatic disease. The pathological type was squamous cell carcinoma for nine patients while one other has a Diffuse large B cell lymphoma. HPV DNA was detected in six cases.

## Conclusion

Ten cases of penile cancer were observed in Cameroon within the AIDS epidemic. These are cases which are confirmed by histology as only 10% of the patients with cancer can have histology performed. The aetiology is unclear. The HIV should be investigated as an etiologic factor.

